# Update on psoriasis immunopathogenesis and targeted immunotherapy

**DOI:** 10.1007/s00281-015-0539-8

**Published:** 2015-11-16

**Authors:** Satveer K. Mahil, Francesca Capon, Jonathan N. Barker

**Affiliations:** St John’s Institute of Dermatology, Division of Genetics and Molecular Medicine, King’s College London, London, UK; Department of Medical and Molecular Genetics, Division of Genetics and Molecular Medicine, King’s College London, London, UK

## Abstract

Over recent years, significant progress has been made in characterisation of the underlying pathogenic mechanisms in psoriasis, a common cutaneous disease that is associated with major systemic co-morbidity and reduced life expectancy. Basic science discoveries have informed the design of novel therapeutic approaches, many of which are now under evaluation in late-stage clinical trials. Here we describe the complex interplay between immune cell types and cytokine networks that acts within self-perpetuating feedback loops to drive cutaneous inflammation in psoriasis. Genetic studies have been pivotal in the construction of the disease model and more recently have uncovered a distinct aetiology for rare, pustular variants of psoriasis. The translation of mechanistic insights into potential advancements in clinical care will also be described, including several treatments that target the interleukin-23 (IL-23)/T17 immune axis.

## Introduction

The therapeutic armamentarium for psoriasis has expanded over the past two decades with the development of several highly selective therapies that are both efficacious and have a favourable safety profile. Novel insights into psoriasis immunopathogenesis have informed the design of these treatments, and in turn, mechanistic studies within clinical trials are helping to further characterise the role of different cellular players and cytokine axes in the pathogenic disease model.

Psoriasis is a phenotypically heterogeneous, immune-mediated skin condition that often follows a relapsing and remitting course. It is a common, complex trait that affects approximately 2 % of the general population and is associated with multiple co-morbidities including arthritis, cardiovascular disease, obesity, hypertension, diabetes mellitus, reduced quality of life and depression [1–4]. Almost 90 % of individuals have psoriasis vulgaris and the majority of research to date (as described in this review) has investigated this form of the disease. It is characterised by well-demarcated, scaling, erythematous plaques that frequently manifest at sites of trauma (extensor aspects of elbows, knees), however can appear anywhere on the body. Approximately one third of patients have moderate to severe disease, which affects more than 10 % of body surface area, and usually necessitates systemic medications. Other clinical variants include pustular psoriasis, guttate psoriasis and erythroderma. Emerging evidence indicates that the distinct phenotypes have different immunogenetic profiles, which will likely influence treatment choices [5]. Discoveries from genetics and immunology research have converged to shape the current pathogenic model for psoriasis. In particular, hypothesis-free large-scale case–control genetic analyses such as genome-wide association studies (GWAS) have highlighted key roles for the regulation of specific innate and adaptive immune pathways, such as antiviral responses and the IL-23/T17 axis, respectively, which have been substantiated by immunological data [6].

## Pathogenic model for psoriasis

The pathogenesis of psoriasis involves dynamic interactions between multiple cell types and numerous cytokines in response to triggers, culminating in the disruption of skin immune homeostasis in genetically predisposed individuals. The histological features of a psoriatic plaque provide an insight into the immunological complexities of the disease. There is thickening of the epidermis (acanthosis) due to an increase in keratinocyte turnover [7]. The retention of keratinocyte nuclei in the stratum corneum (parakeratosis) due to abnormal differentiation further highlights the importance of these skin cells in the development of psoriasis. Psoriatic lesions are also densely infiltrated by T cells and dendritic cells (DC). These immune effectors produce pro-inflammatory cytokines such as tumour necrosis factor α (TNFα), interferon γ (IFNγ), interleukin-17 (IL-17), IL-22, IL-23 and IL-1β. Neutrophils collect in the epidermis and form collections called Munro’s microabscesses. Plaques are highly vascular and new vessel formation is mediated by angiogenic factors such as vascular endothelial growth factor (VEGF).

**Fig. 1:**
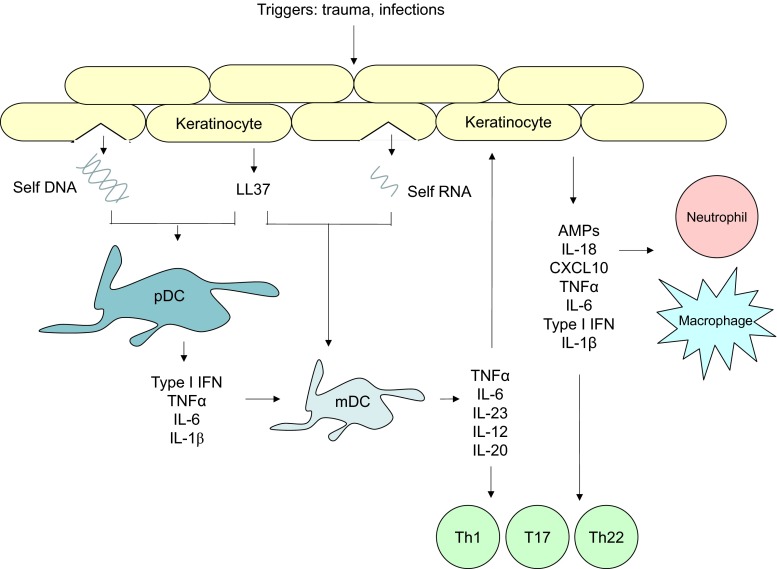
Schema for the initiation of a psoriatic skin lesion. Triggers such as trauma and infections lead to the release of self-DNA and self-RNA, which form complexes with LL37 and activate plasmacytoid dendritic cells (pDCs) and myeloid dendritic cells (mDCs), respectively. pDCs secrete type I interferons (IFN) and other cytokines including TNFα, IL-6 and IL-1β, which promote the activation of mDCs. These antigen presenting cells release pro-inflammatory cytokines that drive T cell-mediated inflammation and keratinocyte activation and proliferation. This promotes the recruitment and activation of further inflammatory cells such as neutrophils and macrophages, contributing to the formation of an inflamed cutaneous plaque. AMPs antimicrobial peptides

The initiation phase of a psoriatic lesion involves a close interplay between external factors and genetic alterations that predispose to the phenotype [3]. Triggers include physical injury (which causes Koebner phenomenon), infections (particularly streptococcal) and medications (e.g. β-blockers, lithium). Although the exact mechanisms for the induction of psoriasis are not yet fully elucidated for many of these environmental factors, some insults such as physical trauma cause the release of the antimicrobial peptide LL37 (cathelicidin) by keratinocytes, which then mediates the breakdown of tolerance to self-nucleic acids (Fig. 1). LL37 binds with pathogen-derived DNA or self-DNA that has been released by stressed or dying cells and forms complexes that activate Toll-like receptor 9 (TLR9) on plasmacytoid DCs [8, 9]. This promotes type I IFN release, which, along with TNFα, IL-6 and IL-1β, activates local myeloid DCs, thus promoting T cell-mediated inflammation. There is also evidence that LL37 may directly activate auto-reactive circulating T cells, and this phenomenon was more prevalent in psoriasis patients with greater disease activity [10].

**Fig. 2:**
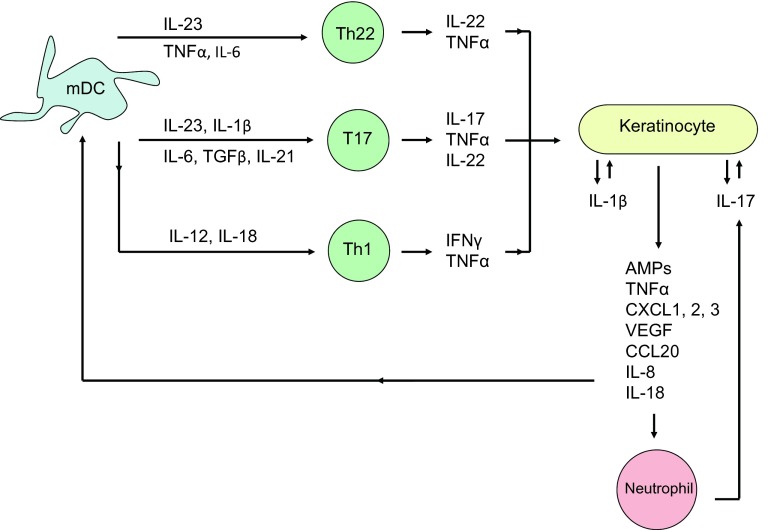
Schema of the contribution of T cell subsets to the pathogenesis of psoriasis. Activated myeloid dendritic cells (mDC) release cytokines that promote the differentiation of populations of resident T cells into Th22, T17 and Th1 cells. Cytokines secreted by these effector T cells stimulate keratinocytes, which promote the recruitment of other inflammatory cells such as neutrophils by release of chemokines. Activation of autocrine and paracrine feedback loops culminates in the development and maintenance of cutaneous inflammation. AMPs antimicrobial peptides, VEGF vascular endothelial growth factor

Myeloid DCs migrate into draining lymph nodes and release cytokines including TNFα, IL-23 and IL-12 that activate allogeneic T cells (Fig. 2). Once activated, T cells enter the circulation and move towards inflamed skin through interactions with adhesion molecules (including P-selectin and E-selectin) on the endothelial cells of blood vessels. The effector molecules secreted by T cells then activate keratinocytes, resulting in the release of cytokines and chemokines that continue to recruit and activate inflammatory cells. For example, IFNγ, IL-17 and IL-22 are secreted by T helper type 1 (Th1), Th17 and Th22 cells, respectively, which contribute to the amplification of cutaneous inflammation.

LL37 may also bind to self-RNA and directly activate myeloid DCs via TLR7 and TLR8 [11]. This results in the upregulation of TNFα and IL-6. In support of this disease initiation model, the TLR7/8 agonist imiquimod has been shown to induce psoriasiform skin inflammation in mouse models [12]. These changes were blocked in mice deficient for the IL-23 or IL-17 receptor, which indicates a role for crosstalk between keratinocytes and the IL-23/T17 pathway in the pathogenesis of psoriasis.

The following sections summarise the roles of specific cells and cytokines in initiating and maintaining the dysregulated immune response that leads to psoriasis. An update on the therapeutic agents currently available and in clinical trial stage is also included.

## The role of immune cell types in psoriasis

### Dendritic cells

DCs are professional antigen presenting cells that activate T cells and are an important source of pro-inflammatory cytokines and chemokines in psoriasis. Genetic studies indicate a fundamental role for antigen presentation in the disease process since the *PSORS1* interval on chromosome 6p21.3 confers the greatest risk and is the most replicated locus for psoriasis [13–15]. The likely causal allele within *PSORS1* is *HLA-Cw6*, which encodes a class I major histocompatibility complex (MHC) molecule that is expressed by antigen presenting cells and mediates T cell activation [16, 17]. It is estimated to account for approximately 50 % of disease heritability and the odds ratios observed in GWAS have ranged between 2.6 and 4.7 [18–21]. Genetic variants within *ERAP1* interact with *HLA-Cw6* (genetic epistasis), such that *ERAP1* risk alleles are only associated with psoriasis susceptibility in individuals harbouring *HLA-Cw6* [19]. The protein product of *ERAP1* trims peptides to enable effective loading onto MHC class I molecules, thus reinforcing the role for antigen presentation and subsequent abnormal T cell activation in the disease model.

The main lineages of DCs that have been characterised in psoriasis are plasmacytoid DCs and myeloid DCs. They localise to the dermis and express distinct cell surface markers. The α_x_ integrin CD11c is a marker of myeloid DCs, which are considered to be important in the early stages of disease. The blood DC antigens (BDCA) identify different subsets of myeloid DCs, such as BDCA-1+ ‘resident’ DCs and BDCA-1− ‘inflammatory’ DCs. The latter have been found in greater numbers in the dermis of lesional psoriatic skin compared with non-lesional or normal skin [22–24] and decrease in number following effective psoriasis treatment [24, 25].

Plasmacytoid DCs are a rich source of type I IFN, an early signature cytokine in psoriasis, and have been found at increased levels in lesional skin compared with normal skin [26–28]. Plasmacytoid DCs help to initiate disease and are activated by LL37/DNA complexes, as described above. The importance of this cell type in the disease model has been supported by xenotransplantation models of psoriasis, in which non-lesional skin from patients with psoriasis are grafted onto athymic nude mice (deficient in T cells) or those with severe combined immunodeficiency (without T and B cells) [29]. In these experimental systems, inhibition of type I IFN release or signalling by plasmacytoid DC blocked pathogenic T cell activation, which prevented the development of psoriasis [15, 16].

Langerhans cells are skin-resident immune cells that associate closely with keratinocytes in the epidermis via E-cadherin. Although Langerhans cells are able to present antigens to T cells in local skin-draining lymph nodes, their role in disease pathogenesis and the nature of the putative psoriasis antigen remains unclear. Previous studies have shown that Langerhans cell migration in non-lesional skin is impaired in early-onset (before age 40; type I) psoriasis [30, 31] and restored with anti-psoriatic biologic treatments [32]. This suggests that loss of cell mobility may cause a dysregulated cutaneous immune response.

### Keratinocytes

Keratinocytes are believed to be crucial in both the early stages of disease pathogenesis and later amplification of chronic inflammatory circuits. As the major constituent of the epidermis, keratinocytes have structural and immunological functions. They form the body’s first line of defence against exogenous physical, chemical and microbial insults. Genetic studies indicate a role for skin barrier dysfunction in psoriasis since deletion of *LCE3B* and *LCE3C* genes, encoding stratum corneum proteins involved in terminal differentiation of the epidermis, was found to be associated with psoriasis [33]. It is hypothesised that incomplete repair after minor skin injury, due to *LCE* gene deletion, contributes to the development of chronic inflammation [34].

Injury to the skin, resulting in cell death, causes the release of antimicrobial peptides (AMPs) by keratinocytes. AMPs, such as LL37, S100 proteins and β-defensins, are key mediators of the innate immune response and have been implicated in psoriasis pathogenesis. Specifically, genetic studies have demonstrated an association between increased β-defensin genomic copy number and risk of disease [35, 36]. AMPs have been shown to be upregulated in psoriasis and its expression is reduced after successful treatment with systemic agents [37]. These molecules have direct antimicrobial activity and also help to modulate immune cells by promoting the upregulation of pro-inflammatory cytokines such as IL-6 and IL-10 and chemokines such as IL-8 (CXCL8) and CXCL10. This mediates the recruitment of macrophages and neutrophils.

In addition to being a rich source of AMPs, keratinocytes also release IL-1 family cytokines including IL-1β and IL-18, which help to initiate the cutaneous inflammatory response to injury. Keratinocytes contain inflammasomes, which are multi-protein complexes that consist of caspase-1, the adaptor protein ASC and a sensor protein (either a nod-like receptor e.g. NLRP3 or a pyrin and HIN domain protein e.g. AIM2), that detect sterile stressors and pathogens [38]. Activated caspase-1 cleaves pro-IL-1 and pro-IL-18 into the mature, active forms of the cytokines. IL-1β thus released has several paracrine effects including the production of TNFα by local keratinocytes and upregulation of leucocyte chemotactic proteins e.g. selectins, which promote the skin infiltration and activation of T cells. IL-18 and IL-1β are further involved in the differentiation of Th1 cells and Th17 cells, respectively (Fig. 2) [39, 40]. This sets up positive feedback loops as activated Th1 and Th17 cells release IL-22 and IL-17 (Th17 only), which drives keratinocyte proliferation and activation, hence contributing to the formation of a cutaneous plaque. T cells also upregulate S100 proteins in keratinocytes, which in turn mediates further leucocyte chemotaxis.

Several genes expressed within keratinocytes that promote innate responses to viral nucleic acids and are upregulated in psoriatic skin lesions have been found by GWAS to confer disease susceptibility. The RIG-I and MDA5 innate antiviral receptors, encoded by the psoriasis-associated genes *DDX58* and *IFIH1*, respectively, bind to viral double-stranded RNA and promote the release of pro-inflammatory cytokines that have been implicated in psoriatic lesion initiation such as type I IFN, IL-1, IL-6, and IL-29 [19, 21, 41]. IL-29 signals through a receptor encoded by *IL28RA*, which has also been identified by GWAS [19]. The signalling cascade triggered by RIG-I and MDA5 is regulated by the protein product of the disease susceptibility gene *RNF114* [42, 43]*.* Hence, dysregulated antiviral immunity may contribute to the development of psoriasis by promoting the over-production of pro-inflammatory cytokines by keratinocytes.

Keratinocytes also produce VEGF during inflammatory states, which induces angiogenesis by promoting the migration, survival and proliferation of endothelial cells, resulting in the formation of an erythematous, vascular plaque. In support, VEGF overexpression in mouse skin results in a psoriasiform phenotype [44].

### Neutrophils

Neutrophils are important in the early stages of psoriasis as they are involved in the recruitment and activation of T cells and the proliferation and differentiation of keratinocytes [45]. Neutrophils are predominantly found in the epidermis in psoriasis and are recruited by the chemokines CXCL1, CXCL2, IL-8 and IL-18. Activated neutrophils release pro-inflammatory cytokines such as IL-17, AMPs and proteases. AMPs are released as neutrophil extracellular traps (NETs), which are web-like structures that are composed predominantly of neutrophil DNA. NETs have been shown to mediate autoimmune disease-associated organ damage [46] and have been identified in psoriatic lesions [47]. Elastase is a protease that is released by neutrophils in response to TNFα or IL-8. It has several downstream effects, including the induction of keratinocyte proliferation and cleavage of cytokines into their active forms [48]. The importance of neutrophil-keratinocyte crosstalk in early psoriasis pathogenesis was highlighted by a recent report [49], in which the anti-IL-17A antibody secukinumab caused a rapid reduction in cutaneous neutrophils alongside histological improvement in keratinocyte abnormalities and downregulation of keratinocyte-derived neutrophil chemoattractants e.g. CXCL1.

### Macrophages

The role of macrophages in psoriasis is not yet fully characterised; however, they are speculated to contribute to the disease as there is a threefold increase in cell numbers in lesional skin, with evidence of normalisation after successful treatment [24]. Mouse models with skin phenotypes that resemble human psoriasis support involvement from skin macrophages in disease development and maintenance [50, 51]. Cutaneous macrophages can be identified by their expression of CD163, and activated macrophages produce high levels of TNFα and are likely to regulate angiogenesis via the release of VEGF [52].

### T cells

**Fig. 3:**
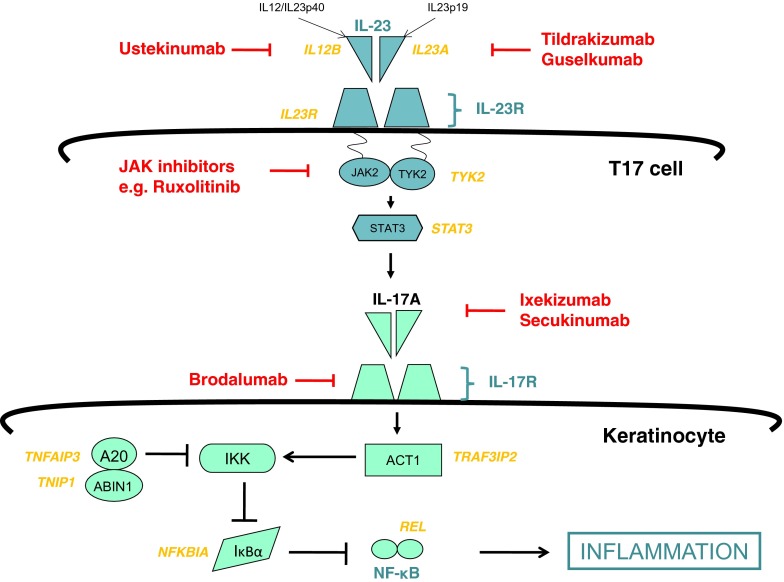
The IL-23/T17 pathogenic axis is an important therapeutic target in psoriasis. IL-23 is a heterodimeric cytokine that is released by dendritic cells and binds to the IL-23 receptor (IL23R) on T17 cells. IL-23R is associated with Jak2 and Tyk2, which activate STAT3 molecules, resulting in the upregulation of IL-17A. Engagement of IL-17R on keratinocytes with IL-17A homodimers or IL-17A/IL-17F heterodimers induces the activation of NF-κB dimers, which translocate to the nucleus and drive the transcription of pro-inflammatory cytokines, chemokines and antimicrobial peptides. Numerous genes (*yellow*) encoding proteins involved in the IL-23/T17 pathway have been shown by genome-wide association studies to confer psoriasis susceptibility. Following activation of IL-17R, ACT1 (encoded by *TRAF3IP2*) interacts with TRAF proteins and the IκB kinase complex (IKK). IKK subsequently phosphorylates the inhibitory proteins IκB (IκBα is encoded by *NFKBIA*), which normally form cytoplasmic complexes with NF-κB. Once phosphorylated, IκB is subject to ubiquitin-induced proteasomal degradation, resulting in the nuclear translocation of NF-κB. Further, the protein products of *TNFAIP3* and *TNIP1*, A20 and ABIN1, respectively, physically interact to enable the ubiquitin-mediated destruction of NEMO (a regulatory protein that activates IKK). Several medications for psoriasis (*red*) target components of the IL-23/T17 immune axis

The importance of abnormal T cell activation in the pathogenesis of psoriasis has been highlighted by several genetic studies that demonstrate a strong disease association with the *HLA-Cw6* allele, as described above. GWAS have also identified multiple genes involved in the IL-23/T17 axis e.g. *IL23R*, *IL23A* and *IL12B* (Fig. 3) [18, 53], which emphasises this particular T cell subset as being crucial to the disease process. Further, a missense mutation (R381Q) in *IL23R* was shown to impair IL-23-induced Th17 activation and effector function and confer protection against psoriasis [54]. Hence, aberrant IL-23 signalling and Th17 activity contribute to chronic inflammation in psoriasis.

A key role for T cells is also indicated by their prevalence in lesional skin biopsies [55]. This is supported by the effectiveness of several T cell-directed therapies in causing disease resolution. The first successful drug was DAB_389_IL-2, an IL-2 fusion protein that causes apoptosis of activated T cells i.e. cells expressing functional IL-2 receptors [56]. The observed beneficial effects of other agents such as abatacept (CTLA-4-Ig), which blocks T cell co-stimulation, and alefacept, an LFA-3-Ig fusion protein that inhibits effector memory T cell activation, further re-enforced the important pathogenic activity of this cell type in psoriasis [57–59]. Clinical improvements with these agents were associated with a decrease in the number of T cells and DCs infiltrating skin lesions. Xenotransplantation mouse models provided additional evidence, since asymptomatic skin grafts developed typical features of psoriasis after injection of activated immunocytes [60]. IL-23-specific monoclonal antibodies prevented such lesions from developing, highlighting the pathogenic importance of Th17 cells [61].

Multiple T cell subsets, each producing a distinct range of cytokines, have been characterised that are relevant to the disease process, including CD4+ Th1, Th17 and Th22 that produce IFNγ/TNFα, IL-17/IL-22 and IL-22, respectively (Fig. 2) [62]. Naive CD4+ T cells differentiate into Th1 cells in the presence of IL-12 [63]; lineage specification of Th17 cells is regulated by IL-6, IL-1β and TGF-β [64, 65] and Th22 cells require TNFα and IL-6 [66, 67]. Subsequent exposure to IL-23 and IL-21 promotes the activation and proliferation of mature, inflammatory Th17 cells [65]. Since there are CD8+ T cells that produce the same cytokines as CD4+ Th17 cells, the term ‘T17 cells’ has been used to encompass all IL-17-producing cells, which also includes T cells expressing the non-variant γδ T cell receptor [68, 69]. Psoriatic skin lesions have greatly increased numbers of γδ T cells compared with healthy controls, and an IL-17-producing γδ T cell population has been identified in the dermis, which may be highly relevant in disease pathogenesis [69, 70].

## The role of cytokines in psoriasis

### TNFα

TNFα is produced by several different cells types in the context of cutaneous inflammation, including macrophages, keratinocytes, Th1 cells, T17 cells, Th22 cells and BDCA-1− inflammatory DCs [71, 72]. Although parts of the literature are conflicting [73], there is evidence that circulating levels of TNFα (in addition to IFNγ, IL-12) are elevated in psoriasis and correlate with disease severity [74, 75].

TNFα regulates the ability of antigen presenting cells such as DCs to activate T cells [76]. It induces the expression of C-reactive protein (part of the acute phase response), several cytokines such as IL-6 (which mediates T cell proliferation and keratinocyte hyperproliferation), and chemokines including CCL20 (recruits myeloid DCs and T17 cells) and IL-8 (for recruitment of neutrophils). Through the upregulation of intercellular adhesion molecule-I (ICAM-1), TNFα promotes the infiltration of inflammatory cells such as T cells and monocytes into the skin. It also facilitates IL-23 production by DCs and enhances the effects of other cytokines relevant to psoriasis pathogenesis such as IL-17. Therefore, TNF antagonists mediate part of their effect via suppression of the IL-23/T17 axis [24].

TNFα has a broad range of effects since TNF receptors (TNFR) are expressed on multiple cell types. There are two types of receptors, TNFR1 and TNFR2. Whereas TNFR2 is expressed predominantly on endothelial and haematopoietic cells, TNFR1 is present on nearly all cell types [77]. Once activated by engagement with TNFα, TNFR modulate multiple aspects of cell function such as proliferation, survival, activation, differentiation and apoptosis, by activating signalling cascades involving NF-κB, mitogen-activated protein kinase (MAPK) and c-Jun N-terminal kinase [78, 79]. Although TNFα blockade is very effective therapeutically, which supports its role in disease pathogenesis, the diverse actions of the cytokine have resulted in numerous drug-associated side effects. Therefore, more targeted immunotherapies are now being investigated.

### IFNγ

In addition to TNFα, Th1 cells are a key source of IFNγ, which is a type II IFN. It is also secreted by DCs and natural killer (NK) cells. Signal transducer and activator of transcription (STAT) 1 is activated downstream of IFNγ and this regulates many genes that are found to be expressed in psoriatic skin lesions [80]. RNA microarrays have demonstrated that a large number of IFNγ-related genes are differentially regulated in psoriasis [81]. However, it was shown that antagonism of IFNγ using a humanised monoclonal antibody does not significantly improve psoriasis [82]. Further, in a clinical trial of an IL-23-specific monoclonal antibody, there was no effect on *IFNG* expression in patients with psoriasis despite a complete clinical and histologic response, in contrast to the significant reduction in *IL17* messenger RNA levels observed [83]. This suggests that IFNγ is not critical in sustaining chronic psoriasis lesions.

It is instead postulated that IFNγ is more relevant in the early stages of disease, through the activation of antigen presenting cells [84]. It promotes the release of IL-1 and IL-23 from DCs, which in turn drives T17 and Th22 cell differentiation and activation. IFNγ also stimulates chemokines (e.g. CXCL10, CXCL11) and adhesion molecule release from keratinocytes, thus facilitating the recruitment of lymphocytes to inflammatory plaques. Although it is known to have an anti-proliferative effect on keratinocytes, this effect is abrogated in psoriatic lesions via the upregulation of suppressor of cytokine signalling (SOCS) 1 in response to high levels of IFNγ [85].

### Type I IFN

Type I IFNs comprise IFNα and IFNβ, amongst others [86]. Several observations have indicated an important role for these cytokines in psoriasis development, particularly in the early stages. Treatment with type I IFN for conditions such as hepatitis and multiple sclerosis has been shown to exacerbate existing psoriasis vulgaris and induce new lesions [87, 88]. The type I IFN signalling pathway is activated in lesional keratinocytes and patients have abnormal serum levels of IFNs [89, 90]. In further support, an increase in IFNα level in xenograft mouse models precedes the development of psoriatic changes and anti-IFNα antibodies block classical psoriatic skin changes such as T cell infiltration into plaques [28].

As discussed above, plasmacytoid DCs, which infiltrate psoriatic skin lesions, are a major source of type I IFN [28] and this promotes myeloid DC phenotypic maturation and activation, thus facilitating T cell priming. Type I IFN signalling modulates the production of IFNγ and IL-17 [91, 92] and has been implicated in the differentiation and activation of T cells, in particular Th1 and T17 cells [93]. Thus, it may drive downstream inflammatory circuits, leading to keratinocyte hyperproliferation. In addition to the indirect modulation of T cell responses via regulation of DCs, type I IFN may have direct pro-survival and pro-proliferative effects on T cells [94]. Finally, type I IFNs are rapidly induced in many different cell types in response to viral infections. Since genetic studies have indicated the importance of innate antiviral immune responses in psoriasis pathogenesis, this also underlines type I IFN as a critical disease cytokine. Specifically, several genes regulating type I IFN production (e.g. *DDX58*, *IFIH1*, *RNF114*) and signalling (e.g. *TYK2*) have been associated with disease susceptibility in GWAS.

### IL-23

IL-23 is a heterodimer that is composed of an IL-23p19 subunit (encoded by *IL23A*) and IL-12/IL-23p40 (shared with IL-12 and encoded by *IL12B*) (Fig. 3). It binds to IL-23R, which is associated with Jak2 and Tyk2. Engagement of the receptor triggers a signalling cascade that involves activation of STAT3. IL-23 is released by DCs and macrophages and mediates the terminal differentiation and activation of T17 cells (including induction of IL-17A and IFNγ), activation of keratinocytes and upregulation of TNFα expression in macrophages. Genetic studies that link single nucleotide polymorphisms in/near *IL-23R*, *IL23A*, *IL12B, TYK2* and *STAT3* with psoriasis susceptibility have highlighted IL-23 as a critical cytokine in disease pathogenesis [18–21]. In support, psoriasis lesions have elevated levels of IL-23 expression [95] and this is reversed after successful treatment with medications such as etanercept [96] and alefacept [97]. Further, anti-IL-12/IL-23 and anti-IL-23 agents are highly effective therapeutic agents [98]. Evidence from mouse models, in which psoriasiform histological changes arise from intradermal injection of IL-23 or overexpression of IL-12/IL-23p40 in keratinocytes, also indicate the importance of this cytokine [99].

### IL-17A

IL-17A belongs to the family of pro-inflammatory cytokines that comprises IL-17A-F [100]. It is overexpressed in psoriasis (both skin and blood [74, 101]) and its involvement in the immunopathogenesis of psoriasis has been increasingly recognised [102]. Given that IL-17 may promote the development of cardiovascular diseases [103], and the established link between psoriasis and such co-morbid conditions, targeting of IL-17 therapeutically may have benefits beyond the sole attenuation of skin inflammation. However, the biological effects of IL-17A in various tissues are complex. Indeed, it may also help to stabilise atherosclerotic plaques [104], which emphasises the need to enrol patients receiving IL-17 inhibitors in long-term safety registries.

Lesional psoriatic T cells produce large amounts of IL-17A when activated ex vivo; however, T cells from healthy skin do not produce IL-17A with the same stimuli [105]. Analysis of the psoriasis transcriptome also reveals enrichment for IL-17A genes [106]. More recently, IL-17A blocking agents have been shown to have rapid and high efficacy in clinical trials, as described later, further emphasising the pathogenic role of IL-17A signalling in psoriasis [107–109].

IL-17A is produced by T17 cells, neutrophils, mast cells and NK cells. Keratinocytes are the predominant cells that express IL-17 receptors (IL-17R; likely consisting of two IL-17RA subunits complexed with one IL-17RC subunit) in psoriasis [110]. The active form of IL-17A consists of either IL-17A homodimers or IL-17A-IL-17F heterodimers; the former having greater biological activity. Engagement of IL-17R induces the activation of NF-κB signalling. GWAS have implicated several genes encoding components of the NF-κB pathway in psoriasis susceptibility including *TNFAIP3*, *TNIP1*, *NFKBIA*, *REL* and *TRAP3IP2* (Fig. 3) [18–21]. For example, a loss of function coding variant in *TRAP3IP2* is associated with psoriasis [20]. *TRAP3IP2* encodes ACT1, which is involved in IL-17 signalling, and Act-1-deficient mice demonstrate upregulated T17 cell responses and spontaneous skin inflammation [111]. This underscores the immunological insights that can be gained from genetic data.

The downstream expression of a large number of genes in response to IL-17A has been shown in a three-dimensional human epidermis model (419 gene probes upregulated and 216 gene probes downregulated) [112]. Keratinocytes are stimulated by IL-17A to produce AMPs; pro-inflammatory cytokines such as IL-19 (driving epidermal hyperplasia), IL-1, IL-6, and IL-23; and chemokines such as IL-8. In addition to promoting the mobilisation and activation of neutrophils, IL-8 is also a chemotaxin for T cells and NK cells. Although the role of regulatory T cells in the pathogenesis of psoriasis remains to be fully elucidated, IL-6 is thought to render effector T cells refractory to regulatory T cell-mediated suppression [113]. IL-17A also increases production of the chemokine CCL20 [114, 115] and ICAM-1, which facilitate cutaneous recruitment of DCs and T cells.

Taken together, IL-17A is crucial to establishing positive feedback loops such that epidermal hyperplasia and the cutaneous inflammatory response are sustained and amplified. For example, recruited DCs may secrete more IL-23, which promotes further T17 cell activation and hence release of IL-17A. This influences keratinocytes, leading to the recruitment of more DCs and T cells to the inflamed skin. IL-17 has recently been shown to act in synergy with TNFα to induce pro-inflammatory cytokine production by keratinocytes [115]. Indeed, genes that are synergistically regulated by IL-17 and TNFα were more effectively blocked by anti-IL-17A than TNF antagonists [102], suggesting that IL-17A may have a dominant pathogenic effect.

### IL-22

IL-22 is a member of the IL-10 family of cytokines and has been found to be upregulated in the skin and sera of patients with psoriasis [116, 117]. Expression is also reduced following anti-psoriatic therapies [117]. The production of IL-22 by Th22 cells and Th17 cells is induced by IL-23 and it mediates multiple effects on keratinocytes, including hyperproliferation, differentiation, migration, and pro-inflammatory cytokine and AMP production [118, 119]. IL-22 has been shown to act in synergy with IL-17A to induce AMP production by keratinocytes [120]. Blockade of IL-22 in vivo or genetic deletion caused reduced IL-23-induced epidermal hyperplasia [121], and IL-23-mediated epidermal hyperplasia in a murine model of psoriasiform skin inflammation was found to be dependent on IL-22 [121]. These data highlight potential crosstalk between the IL-23/T17 pathway and IL-22/Th22. However, in contrast to the IL-23/T17 pathway, there is a lack of genetic data in support of a role for IL-22 in disease pathogenesis. Further, trials of a human monoclonal antibody targeted against IL-22 (fezakinumab) were discontinued since preliminary analyses showed that the efficacy endpoints could not be achieved [122]. The negative findings from both genetics and clinical studies suggest that IL-22 may not be as critical to the disease process as had initially been anticipated from earlier immunological studies.

## Pustular psoriasis

Pustular psoriasis is a rare, severe subtype of psoriasis that has been shown by genetic studies to have a distinct aetiology from psoriasis vulgaris. In particular, a lack of association of pustular psoriasis with the *PSORS1* locus has been demonstrated, in striking contrast to psoriasis vulgaris [123]. It is characterised clinically by the presence of sterile pustules on variably erythematous skin and histologically by diffuse dermal neutrophilic infiltration and micropustules in the epidermis [124, 125]. It encompasses generalised pustular psoriasis, in which patients experience acute flares of widespread cutaneous pustulation associated with systemic upset, and chronic, localised forms such as palmoplantar psoriasis and acrodermatitis continua of Hallopeau.

Recently, IL-1 family cytokines have been shown to have a potential pathogenic role in pustular psoriasis since loss of function, autosomal recessive mutations in *IL36RN* were described in association with this disease subtype [126–128]. Targeted sequencing studies further revealed that mutations in *IL36RN* are not associated with psoriasis vulgaris, emphasising distinct pathogenic mechanisms for pustular and plaque forms of psoriasis and the potential for stratification of psoriasis subtypes using genetic biomarkers [129]. *IL36RN* encodes an antagonist (IL-36Ra) that blocks innate immune IL-1 family cytokines (IL-36α, IL-36β and IL-36γ) from binding to their receptor (IL-1RL2) [130]. This prevents subsequent activation of the NF-κB pathway. Therefore, IL-36Ra deficiency leads to unopposed IL-1 activity that may result in the significant cutaneous neutrophil recruitment that is observed in pustular psoriasis. IL-36 cytokines also cause upregulation of IL-23 by DCs and keratinocytes [131], IL-6 and IL-8, which helps to sustain cutaneous inflammation.

In further support of a role for aberrant IL-1 signalling in pustular psoriasis, the disease is associated with pathogenic mutations in *AP1S3*, silencing of which has been shown to disrupt the endosomal translocation of Toll-like receptor 3 (TLR3), leading to impaired IFNβ induction [132]. Given that IFNβ downregulates the production of IL-1 [133], it is possible that mutations in *AP1S3*, which encodes a subunit of adaptor protein complex 1 and is involved in clathrin-mediated vesicular transport of proteins between the trans-Golgi network and endosomes, result in IL-1 over-production. By virtue of the aforementioned pathogenic insights delivered by genetic studies, IL-1 blockade is now emerging as a promising therapeutic strategy for this clinical variant.

Pustular psoriasis is also associated with missense mutations in *CARD14* [134–136]. *CARD14* is highly expressed in the skin and encodes a protein involved in TRAF2-dependent NF-κB activation [137]. It has been previously implicated in psoriasis vulgaris [138], which suggests some potential shared disease pathways in distinct subtypes of psoriasis.

## Update on therapeutics

As the pathogenic mechanisms have become better defined, there has been a shift towards the design of more targeted treatments in psoriasis (Table 1). Specific cytokines pertinent to the development of disease have been selected as drug targets in the hope of effective suppression of pathogenic immune responses whilst reducing the risk of global suppression of protective immunity, thus potentially improving the safety profiles of the medications.

**Table 1:** Targeted therapies for psoriasis

Therapeutic agent	Target	Agent type	Stage of development
Infliximab	TNFα	Chimeric monoclonal antibody	Approved
Adalimumab	TNFα	Human monoclonal antibody	Approved
Etanercept	TNFα	Soluble TNFα receptor-IgG fusion protein	Approved
Ustekinumab	IL-12/IL-23p40	Human monoclonal antibody	Approved
Tildrakizumab, guselkumab	IL-23p19	Human monoclonal antibody	Phase III studies ongoing
Ixekizumab	IL-17A	Humanised monoclonal antibody phase III	Studies ongoing
Secukinumab	IL-17A	Human monoclonal antibody	Approved
Brodalumab	IL-17RA	Human monoclonal antibody	Development halted
Apremilast	PDE-4	Small molecule inhibitor	Approved
Tofacitinib	JAK1/JAK3	Small molecule inhibitor	Phase III studies completed; under FDA review
Ruxolitinib	JAK1/JAK2	Small molecule inhibitor	Phase II studies completed
CF101	A_3_ adenosine receptor	Small molecule agonist	Phase II/III studies completed
Anakinra	IL-1R	Soluble recombinant IL-1Ra	Phase II study ongoing
MABp1	IL-1α	Humanised monoclonal antibody	Phase II study completed
Canakinumab	IL-1β	Human monoclonal antibody	Not currently in trial
Gevokizumab	IL-1β	Humanised monoclonal antibody	Not currently in trial

### TNF antagonists

TNF antagonists have proven to be highly effective for the treatment of psoriasis. The three agents currently approved for use in moderate/severe psoriasis are infliximab, a chimeric neutralising monoclonal antibody, adalimumab, a fully humanised IgG1 monoclonal antibody, and etanercept, a recombinant fusion protein comprising an Fc domain of human IgG1 monoclonal antibody and the ligand binding domain of the TNFα receptor. Effective treatment causes decreased numbers of T cells and DCs and reduced levels of their secreted cytokines [24, 139]. In particular, successful therapy was found to be associated with downregulation of genes involved in the differentiation and function of Th17 cells, suggesting that TNF antagonists exert their effect via the modulation of the IL-23/T17 axis. This may be attributable to TNFα promoting IL-23 synthesis in DCs. Etanercept treatment was also shown to reduce lesional DC expression of co-stimulatory molecules in vitro, thus impairing DC-T cell interactions and the activation of allogeneic T cells [24].

However, blockade of TNFα may lead to serious adverse events, including reactivation of latent tuberculosis, and this has prompted more rigorous screening investigations prior to commencing all biologic agents [140]. Some studies have also described an association between treatment and an increased incidence of malignancy such as lymphoma [141, 142]. There are reports of TNF antagonists rarely promoting the development of demyelinating disease, and a potential underlying mechanism has been unravelled through the discovery of a multiple sclerosis-associated genetic variant that translates into the production of an endogenous TNF antagonist called Δ6-TNFR1 [143, 144]. This soluble protein comprises the extracellular domain of TNFR1, but lacks the transmembrane or cytoplasmic domains. It can bind and neutralise TNF with high affinity, thus preventing potentially neuroprotective cellular signalling through membrane-bound TNFR1. Finally, TNF antagonists have been associated with a de novo, paradoxical onset of pustular psoriasis mostly located on the palms and/or soles, for which a mechanism is currently unknown [145].

### IL-12/IL-23 inhibitors

Since the characterisation of a dominant pathogenic role for the IL-23/T17 axis in psoriasis by GWAS, several drugs targeted against components of this pathway have been studied with reported successful outcomes (Fig. 3). Ustekinumab is a Food and Drug Administration (FDA)-approved humanised monoclonal antibody that neutralises the p40 subunit common to IL-23 and IL-12. The antibody prevents the binding of IL-23 and IL-12 to their receptors, thus inhibiting T17 and Th1 signalling pathways. It has been shown to be a highly efficacious treatment, with greater than 60 % of treated patients achieving at least 75 % reduction in their baseline Psoriasis and Severity Index (PASI-75) at 12 weeks compared with 3 % of the control group [146, 147]. There is also a reported superior clinical effect compared with etanercept [148], suggesting that IL-23 may have a more prominent role than TNFα in psoriasis pathogenesis. Indeed, IL-23 levels remain high in patients who fail TNF antagonists, which enable ongoing T17 activation [139]. Although limited follow-up data is available, the safety profile of ustekinumab to date appears to be more favourable than TNF antagonists, which may be due to the intact TNFα-mediated innate immune responses that result from IL-23 antagonism.

There are emerging reports of successful treatment of different subtypes of pustular psoriasis with ustekinumab [149–152]. However, the case reported of successful treatment of acrodermatitis of Hallopeau, a severe and often refractory form of pustular psoriasis affecting distal fingers and toes, required co-therapy with acitretin and higher than standard doses of ustekinumab in order to achieve complete clinical resolution [151]. Further assessment of this medication within adequately powered clinical trials is thus warranted; however, this is challenging given the lower prevalence of this form of psoriasis.

There are encouraging results from clinical studies of monoclonal antibodies that target the unique p19 subunit of IL-23 in psoriasis vulgaris [153]. Seventy-four percent of patients treated with tildrakizumab, a monoclonal anti-p19 IgG1, achieved PASI-75 after 16 weeks compared with 4.4 % of individuals in the placebo group in a phase II trial. In a study of guselkumab (anti-p19), 81 % of patients achieved PASI-75 after 16 weeks in the treatment group, compared with 71 % of those receiving adalimumab and 4.8 % of those receiving placebo.

### IL-17 inhibitors

IL-17A is a central driver in disease pathogenesis; hence, IL-17 inhibitors have been extensively researched for the treatment of psoriasis. Secukinumab and ixekizumab are neutralising humanised monoclonal antibodies (IgG4 and IgG1, respectively) that bind to IL-17A and brodalumab binds to the IL-17 receptor A subunit.

Secukinumab received FDA approval for the treatment of moderate/severe psoriasis in January 2015. It demonstrated clinical efficacy in phase II trials, with 82 % of treated patients achieving PASI-75 compared with 9 % of those receiving placebo [154]. This agent was shown to be more effective than etanercept in the 52-week randomised, double-blind, placebo-controlled, parallel-group, phase III FIXTURE study, with similar incidences of adverse events [155]. The trial also showed more rapid effects with secukinumab as clinical response (defined as a 50 % reduction in mean PASI) was achieved sooner with secukinumab (median 3 weeks with 300 mg and 3.9 weeks with 150 mg) than etanercept (median 7 weeks). Candidal infections were more common in those treated with secukinumab than etanercept, which is likely attributable to the important role for IL-17A in mucocutaneous immunity against fungi. All candidal infections were, however, either self-limited or resolved with standard treatments and none required cessation of secukinumab. A second randomised phase III trial (ERASURE), comparing secukinumab with placebo, demonstrated superior responses in the treatment group at 12 weeks [155].

Phase II trial data of ixekizumab showed that 82 % of treated patients achieved PASI-75 at 12 weeks with no associated serious adverse events [109]. A rapid clinical response was observed, with many achieving near maximal improvement within the first 6 weeks of treatment, which is faster than that observed with other available therapies, including TNF antagonists. Phase III studies of ixekizumab are ongoing (https://clinicaltrials.gov).

Mechanistic studies have demonstrated decreased expression of a broad range of immune-related genes in response to secukinumab treatment, including T17-related transcripts (*IL22*, *IL17F* and *IL8*), Th1-related genes (*IFNG* and *IL12B*) and other innate immune inflammatory genes (*TNF*, *IL6* and *IL1B*), which may account for the potency of the medication [156]. There also was evidence of decreased epidermal hyperplasia, indicating keratinocyte modulation, and reduced infiltration of CD3+ and IL-17+ cells in lesions. A later study showed a reduction in the inflammatory cell infiltrate (CD3+, CD11c+ and CD-LAMP+ cells) in response to ixekizumab, indicating effects on both T cells and DCs [102]. The medication modulated gene expression rapidly, with normalisation (i.e. reduction by at least 75 %) of 60 % of transcriptome genes relevant to psoriasis after only 2 weeks, compared with 10 % of disease-related genes normalised by etanercept. This study also highlighted the wide-ranging effects of IL-17, since hundreds of psoriasis-associated genes were normalised by ixekizumab.

Brodalumab has a potentially wider range of action as it antagonises the receptor that binds IL-17A, IL-17F and IL-17A/F heterodimers. A phase II clinical trial showed that 82 % of treated patients reached PASI-75 at week 12 [107], and phase III studies demonstrated superior efficacy of brodalumab compared with both placebo and ustekinumab for all primary endpoints [157]. Despite these promising reports, there has been recent concern over potential links between brodalumab and suicidal ideation since two patients enrolled in the published clinical studies committed suicide. Although no causality has been established between these events and brodalumab, it underlines our current relatively limited understanding of the safety profiles of these medications.

### Phosphodiesterase inhibitors

The oral phosphodiesterase-4 (PDE-4) inhibitor apremilast prevents the conversion of 3′-5′-cyclic adenosine monophosphate (cAMP) to AMP. Its beneficial effect is thus attributable to increased levels of cAMP, which reduces inflammation by downregulating cytokines such as TNFα and IL-23. It also upregulates the production of anti-inflammatory molecules such as IL-10 [158]. Phase II and III study data demonstrated superior benefit in the treatment groups compared to placebo, with a favourable safety profile reported, and it was approved by the FDA and European Medicines Agency (EMA) for the treatment of moderate/severe psoriasis in 2014 [159].

### Janus kinase inhibitors

Janus kinases (JAK) are cytoplasmic protein tyrosine kinases that mediate the activation of STAT proteins. JAK/STAT intracellular signalling regulates the expression of pro-inflammatory genes. Numerous cytokines that are upregulated in psoriatic skin lesions and involved in T cell proliferation, activation and survival, such as type I and III IFNs and IL-23, use the JAK/STAT pathway; however, there some exceptions, including TNFα and IL-17. There are four members of the JAK family, JAK1, JAK2, JAK3 and TYK2. JAKs act in pairs and the novel inhibitors that are currently being evaluated in clinical trials have varying efficacy for each of the JAKs. TYK2 is involved in modulation of T17 cell responses, and although there are no selective TYK2 inhibitors currently in clinical trials, the pathogenic missense mutations in *TYK2* discovered by GWAS emphasise its role in disease pathogenesis and the utility of pursuing it as a novel drug target [19].

Tofacitinib is a small molecule that preferentially inhibits JAK1 and JAK3. Phase II study data demonstrated a PASI-75 response in 67 % of patients with moderate/severe plaque psoriasis receiving 15 mg daily [160]. In this study, the side effects included dose-dependent increases in lipids (which returned to baseline after cessation of treatment) and mild decreases in haemoglobin and neutrophil counts. Although small molecules generally have less efficacy when compared with biologic agents, their associated advantages include oral administration (or topical, as below) and reduced cost.

Small molecules less than 500 Da in molecular weight are able to cross the stratum corneum, so may be used as topical treatments [161]. Topical 2 % tofacitinib formulations were well tolerated and showed promising efficacy in a recent vehicle-controlled trial [162]. Ruxolitinib predominantly inhibits JAK1 and JAK2 and topical formulations have previously been tested [163]. This demonstrated a 53 % and 54 % decrease in mean ‘total lesion score’ (assessing scaling, redness and thickness) after treatment with 1 % and 1.5 % ruxolitinib ointments, respectively, compared with 32 % for vehicle. No significant adverse effects were reported.

### A_3_ adenosine receptor agonists

A_3_ adenosine receptors (A_3_AR) are G protein coupled receptors that bind to adenosine. They were found to be highly expressed in peripheral blood mononuclear cells from psoriasis patients [164]. Activation of A_3_AR by the agonist CF101 has been shown to reduce NF-κB signalling and promote apoptosis of inflammatory cells. Pro-inflammatory cytokines such as TNFα, IL-6 and IL-12 are also downregulated. CF101 has subsequently been tested in a phase II clinical trial [165]. Orally administered CF101 (2 mg twice daily) resulted in a PASI-50 response in 35.3 % of patients, with only mild side effects reported.

### IL-1 antagonists

Given the potential importance of dysregulated IL-1 signalling in the pathogenesis of pustular psoriasis, IL-1 blockers have been investigated for use in the treatment of this clinical phenotype, with successful cases described [166–168]. The agent anakinra has been used, which is a recombinant form of the IL-1 receptor antagonist (IL-1Ra) that prevents both IL-1α and IL-1β signalling. However, randomised control trial data is lacking and the reported cases of incomplete clinical response suggest that IL-1 signalling may not play a dominant pathogenic role in all patients with pustular psoriasis [151, 169–171].

## Conclusion

A limitation of the current clinical studies is the lack of long-term data, which is particularly relevant when considering the safety profile of the medications. For example, efalizumab was first approved in 2003 for the treatment of moderate/severe psoriasis but later withdrawn from the market in 2009 due to safety concerns. It is a humanised, monoclonal antibody that blocks the interaction between CD11a/LFA-1 on T cells and ICAM-1 on antigen presenting cells. Efalizumab was withdrawn after progressive multifocal leukoencephalopathy, a potentially fatal central nervous system infection associated with immunosuppression, was reported in four patients receiving treatment [172]. Further, although biosimilar alternatives to biological agents that have lost their patent protection are of considerable financial interest, the importance of critical assessments of their safety, quality and efficacy relative to the biological reference product prior to use in patients must be emphasised [173].

Careful monitoring of all new and existing therapies and recording of data in multi-centre, large-scale registries are vital to ensuring adverse events are promptly recognised. It will also be important for clinical studies to stratify patients according to detailed phenotype and genotype information. This will help to identify biomarkers of drug response and represent a shift towards the practice of personalised medicine, in which an individual is prescribed a specific treatment that is predicted to have therapeutic success and a favourable safety profile, on the basis of their genetic profile.

Although further research is warranted, current clinical trial data on new treatments are helping to improve our understanding of the immunopathogenesis of psoriasis. Indeed, psoriasis research is a leading example of the translational potential of data from basic science studies such as GWAS, which have delivered mechanistic insights into disease pathways. These have subsequently informed the design of powerful new treatments that are able to act faster, more effectively and in a more targeted manner. Improved specificity of drug targets reduces the chance of ameliorating the body’s protective immunity against microbes, which is currently a limitation of agents such as TNF antagonists. Although late-stage clinical trials for several medications are still underway, there appears to be increasing hope for the millions of individuals worldwide suffering from this debilitating and disfiguring disease.
